# Genome Sequence of Campylobacter Strain 19-13652, Isolated from Breeding Pheasants

**DOI:** 10.1128/mra.01184-21

**Published:** 2022-05-16

**Authors:** Arianna Peruzzo, Carmen Losasso, Adriana Di Castri, Ilenia Drigo, Luca Bano, Massimiliano Orsini

**Affiliations:** a Laboratory of Microbial Ecology and Genomics, Istituto Zooprofilattico Sperimentale delle Venezie, Legnaro, Italy; b Laboratory of Clinical Diagnostic and Serology, Istituto Zooprofilattico Sperimentale delle Venezie, Treviso, Italy; c Laboratory of Special Bacteriology, Istituto Zooprofilattico Sperimentale delle Venezie, Treviso, Italy; Loyola University Chicago

## Abstract

We report the whole-genome sequence of a Campylobacter strain that was isolated from breeding pheasants presenting “bulgy eyes” in Italy. Traditional molecular typing methods did not return any reliable result. Whole-genome sequencing and sequence comparison with known genomes did not meet the criteria for assignment to an existing species.

## ANNOUNCEMENT

The Campylobacter genus includes more than 40 species. Among them, Campylobacter coli, Campylobacter jejuni, Campylobacter fetus, Campylobacter hyointestinalis subsp. *hyointestinalis*, Campylobacter lari, and Campylobacter upsaliensis have been isolated from both humans and animals. Moreover, members of the Campylobacter genus are able to cause disease in wild animals. A Campylobacter strain, 19-13652, was isolated from breeding pheasants affected by “bulgy eyes” disease, a common infectious sinusitis in this kind of birds (1). A bacteriological examination was conducted from the infraorbital sinus with severe sinusitis, and a plethora of bacteria were isolated. These bacteria included a small hemolytic colony grown on blood agar for 48 h at 37°C under microaerophilic conditions. Microscopic observation (at ×1,000 magnification) of the Gram-stained hemolytic colony revealed the presence of comma-shaped Gram-negative bacteria compatible with Campylobacter spp. The hemolytic culture was not identified with either commercial miniaturized biochemical kits (API Campy; bioMérieux) or matrix-assisted laser desorption ionization–time of flight (MALDI-TOF) mass spectrometry (MALDI Biotyper Microflex LT; Bruker Daltonics). The DNA was extracted using a QIAmp DNA minikit (Qiagen). One nanogram of genomic DNA was used for preparation of a library for the Illumina platform using the Nextera XT DNA library preparation kit (Illumina). Sequencing was performed on the Illumina NextSeq 550 platform (2 × 150-bp paired-end reads), which returned 936,851 paired-end reads (theoretical coverage, 180×). An additional 1.5 ng was used for the preparation of Nanopore libraries using a SQK-RBK004 rapid sequencing kit, and the libraries were sequenced using a FLO-MIN106 R9 flow cell on a MinION device (Oxford Nanopore Technologies, Oxford, UK), which returned 9,579 long reads (*N*_50_ = 6,824 bp; theoretical coverage = 26.7×). After read cleaning was performed with fastp (2) and Nanofilt (3) for Illumina and Nanopore data, respectively, a preliminary assembly approach was performed using Canu v2.1.1 (4) with default parameters (minReadLength = 1000; minOverlapLength = 500) followed by Pilon (5) for sequence refinement using the Illumina reads. Default parameters were used for all software tools unless otherwise noted. The final assembly consisted of a singular circular sequence of 1,837,327 bp (GC content, 41.0%); it was submitted to the DDBJ Center for annotation using the DFAST annotation pipeline v4.3 (6), which rotates the molecule starting from the *dnaA* gene. No assembled sequences ascribable to plasmids were detected. Annotation returned 1,733 protein-coding genes, 1 pseudogene, 6 rRNA genes, 45 tRNA genes, and no CRISPR arrays. DFAST performs both completeness and taxonomy checks. While the former returned a completeness value of 99.54%, the latter compared the submitted sequence to 15 Campylobacter genomes (GenBank assembly accession numbers GCA_002021925.1, GCA_000736415.1, GCA_002139935.1, GCA_004803795.1, GCA_013372125.1, GCA_004803815.1, GCA_001298465.1, GCA_013201685.1, GCA_012978875.1, GCA_000376325.1, GCA_013372205.1, GCA_012978755.1, GCA_900063025.1, GCA_008802075.1, and GCA_003289475.1) from 11 different species. Comparison was performed using the FastANI tool (7), which returned as the closest match Campylobacter pinnipediorum subsp. *pinnipediorum* strain RM17260 (GenBank assembly accession number GCA_002021925.1), with an average nucleotide identity (ANI) of 78.89%. This finding led us to not consider this strain the same species, since the assumption of strains belonging to the same species involves an ANI value of >95%. Similarly, the average amino acid identity (AAI) calculated by the AAI-profiler tool (7) returned a best value of 68.4% against two Campylobacter curvus strains. Although new species confirmation requires additional assays, the availability of genome sequences for isolates from minor niches improves the knowledge of the ecological, virulence, and genomic diversity of the Campylobacter genus.

### Data availability.

The genome assembly is available in the GenBank database under the accession number AP024713, while raw reads can be accessed at SRA (SRA accession numbers SRR17148446 and SRR17148447). Both raw data and assemblies are under the BioProject accession number PRJNA785762.

## References

[1] Blackall PJ, Hinz KH. 2008. Infectious coryza and related diseases. In: Poultry diseases. WB Saunders. p. 155–159. doi:10.1016/B978-0-7020-2862-5.50016-7.

[2] Chen S, Zhou Y, Chen Y, Gu J. 2018. fastp: an ultra-fast all-in-one FASTQ preprocessor. Bioinformatics 34:i884–i890. doi:10.1093/bioinformatics/bty560. 30423086 PMC6129281

[3] De Coster W, D'Hert S, Schultz DT, Cruts M, Van Broeckhoven C. 2018. NanoPack: visualizing and processing long-read sequencing data. Bioinformatics 34:2666–2669. doi:10.1093/bioinformatics/bty149. 29547981 PMC6061794

[4] Koren S, Walenz BP, Berlin K, Miller JR, Bergman NH, Phillippy AM. 2017. Canu: scalable and accurate long-read assembly via adaptive *k*-mer weighting and repeat separation. Genome Res 27:722–736. doi:10.1101/gr.215087.116. 28298431 PMC5411767

[5] Walker BJ, Abeel T, Shea T, Priest M, Abouelliel A, Sakthikumar S, Cuomo CA, Zeng Q, Wortman J, Young SK, Earl AM. 2014. Pilon: an integrated tool for comprehensive microbial variant detection and genome assembly improvement. PLoS One 9:e112963. doi:10.1371/journal.pone.0112963. 25409509 PMC4237348

[6] Tanizawa Y, Fujisawa T, Nakamura Y. 2018. DFAST: a flexible prokaryotic genome annotation pipeline for faster genome publication. Bioinformatics 34:1037–1039. doi:10.1093/bioinformatics/btx713. 29106469 PMC5860143

[7] Jain C, Rodriguez-R LM, Phillippy AM, Konstantinidis KT, Aluru S. 2018. High throughput ANI analysis of 90K prokaryotic genomes reveals clear species boundaries. Nat Commun 9:5114. doi:10.1038/s41467-018-07641-9. 30504855 PMC6269478

